# A preoperative predictive tool to assess the need for staging laparoscopy in oesophagogastric cancer patients

**DOI:** 10.1308/rcsann.2022.0140

**Published:** 2023-08-29

**Authors:** JM Halle-Smith, T Bage, SK Kamarajah, M Siddaiah-Subramanya, R Pande, JL Whiting, EA Griffiths

**Affiliations:** ^1^ Queen Elizabeth Hospital, University Hospitals Birmingham NHS Foundation Trust, UK; ^2^ Institute of Cancer and Genomic Sciences, University of Birmingham, UK

**Keywords:** Oesophageal cancer, Gastric cancer, Staging laparoscopy

## Abstract

**Introduction:**

Staging laparoscopy (SL) has become commonplace in the preoperative staging pathway for oesophagogastric (OG) cancer. SL is often performed before curative treatment to examine for macroscopic peritoneal metastases (PM) or positive peritoneal cytology (PPC). The aim of this study was to develop an objective risk scoring system to predict both PM and PPC at SL.

**Methods:**

A prospectively collected and maintained database of all OG cancer patients treated between 2006 and 2020 was reviewed. Univariate and multivariate analyses were performed to identify risk factors for both PM and PPC at SL. A risk score was produced for both PM and PPC, and then validated internally.

**Results:**

Among 968 patients who underwent SL, 96 (9.9%) had PM and 81 (8.4%) had PPC at SL. Tumour site (*p* < 0.001), computed tomography (CT) T stage (*p* < 0.001) and N stage (*p* = 0.029) were significantly associated with PM at SL (*p* < 0.001). Tumour site (*p* < 0.001), biopsy histology (*p* = 0.041), CT T stage (*p* < 0.001) and N stage (*p* < 0.001) were significantly associated with PPC. The risk scoring model for PM included cancer site and CT T stage. This was successfully tested on the validation set (area under the receiver operating characteristic [AUROC] = 0.730). The risk scoring model for PPC included cancer site, CT T and N stage. This was successfully tested on the validation set (AUROC = 0.773).

**Conclusions:**

The current risk scores are valid tools with which to predict the risk PM and PPC in patients undergoing SL for OG cancer and may help to avoid subjecting patients to unnecessary SL.

## Introduction

Oesophagogastric (OG) cancers are common^1-3^ and carry a poor prognosis given that the majority present with locally advanced disease, irresectable or metastatic disease so are not amenable to radical therapy.^1-5^ Curative resection, with or without neoadjuvant chemotherapy, remains the mainstay of radical treatment, which is only possible in around 25% of cases. The tendency of both oesophageal and gastric cancer to spread to the peritoneum^2,4,6^ means that accurate preoperative staging is vital to avoid the considerable morbidity and mortality associated with unnecessary attempts at curative resection.^7^ Given that peritoneal carcinomatosis (PC) in these cancers can be undetectable on preoperative cross-sectional imaging in up to 40% of cases,^8^ staging laparoscopy (SL) has become commonplace in the preoperative staging pathway for OG cancer.^2,9^ However, its precise place in the staging pathway is uncertain and some centres perform it routinely, whereas others are more selective.

SL is a reliable method of detecting PC via direct visualisation or peritoneal lavage and cytological examination for the detection of intraperitoneal free cancer cells.^5,10^ It has a low rate of morbidity and is normally a day case procedure.^11,12^ That said, diagnostic yield has been shown to vary considerably between different types of OG cancer, with higher rates of up to 45% seen in advanced gastric cancer^10,11,13^ compared with lower rates of 5%–11% in oesophageal cancer.^9,13,14^ Furthermore, it has been shown that rates of PC are significantly greater in certain patient subgroups, namely those with larger tumours^9-11^ and poorly differentiated adenocarcinoma^2,6,9^ for example, whereas lower rates of PC have been shown in patients with intrathoracic tumours and those with squamous cell carcinoma.^8^ PC may be present in the form of malignant cells found in peritoneal washings (positive peritoneal cytology [PPC]) or macroscopic peritoneal metastases (PM) seen at laparoscopy. The distinction between these is important because at most institutions the treatment of PPC and PM is different. For example, a patient with PPC may still be considered for radical therapy if they responded to chemotherapy and were converted to negative cytology at repeat SL,^15,16^ whereas a patient with PM would be for palliative chemotherapy, stenting or best supportive care depending on circumstances.^4,15,17,18^

As a result, some have questioned the utility of SL in certain groups of OG cancer patients, because for patients with a low diagnostic yield it may unnecessarily prolong time to radical treatment resulting in increased chance of disease progression. Furthermore, while the SARS-CoV-2 (COVID-19) pandemic continues to place strain upon health systems worldwide, operating theatre capacity remains at a premium and during the peaks of the pandemic many units stopped offering SL.^19^ Therefore, the aim of this study is to develop an objective risk scoring system to determine which patients will benefit most from SL during treatment planning and those in whom it is safe to forego SL and proceed straight to curative resection.

## Methods

Given that this was a study focusing on diagnostic accuracy, it was designed and carried out in line with the latest Standards for the Reporting of Diagnostic accuracy guidelines, published in 2015.^20^

### Patient selection

All patients with OG cancer managed by our unit between January 2006 and April 2020 were reviewed using the prospectively collected departmental database. This data set included patient demographics, staging investigations, operative details, oncological treatment, histopathology reports and long-term follow-up with recurrence and survival reported. All patients had SL performed at the Queen Elizabeth Hospital, Birmingham. Our institution receives referrals for OG cancer from across the West Midlands Region, including Walsall Manor Hospital, Russells Hall Hospital, Sandwell Hospital and City Hospital. This study was approved by the institutional audit management system (CARMS – 00103).

### Data collection

Preoperative variables, available at the time of preoperative multidisciplinary team meeting (MDT), were used to develop the risk scores. Cancer site was determined by the tumour location on preoperative investigations and was classified as oesophageal, gastro-oesophageal junction (GOJ) or gastric. Histological diagnosis was obtained from the biopsy taken during oesophagogastroduodenoscopy (OGD). This was classified as adenocarcinoma, squamous cell carcinoma or ‘other’ for the purposes of statistical analysis. Examples of tumour types in the ‘other’ category were lymphoma and sarcoma. The computed tomography (CT) and endoscopic ultrasound (EUS) T and N stage was the staging value given in the report of the investigation. Positron emission tomography (PET) scanning was used for staging patients with oesophageal and GOJ cancers, but was not routinely used for gastric cancer.

### Operative methods

Prior to the COVID-19 pandemic, our unit had offered a SL to patients who were considered suitable for the curative pathway, i.e. all patients who had M0 disease on CT staging who were considered fit for surgical resection. The only exceptions were patients with previous laparotomy in whom it was thought that laparoscopy would not be feasible or risk injury. PET was used to stage oesophageal and GOJ tumours but not purely gastric tumours, and typically this was done before laparoscopy. Laparoscopy was performed using a three-port technique. Abdominal viscera were examined in a systematic fashion as described elsewhere^21^ and any suspicious peritoneal, omental or liver lesions were biopsied for histology. Peritoneal lavage was performed routinely and this was done by introducing 150ml of warm 0.9% saline into the peritoneal cavity (before any biopsy). This was stimulated externally for 3min, then aspirated from the pelvis and/or subphrenic region. Cytology specimens were processed by centrifugation (3,000r.p.m.) for 5min, followed by either direct smearing or a further cytospin, depending on sample density. Four slides were prepared for each patient, fixed in alcohol and stained using Papanicolaou methods. All slides were analysed by one of two consultant cytopathologists, and patients were deemed to have positive cytology if the slides unequivocally demonstrated the presence of free malignant cells. In indeterminate cases, immunohistochemistry was used. All patients were discussed in the upper gastrointestinal cancer MDT.

### Statistical analysis

Initially, the patient group was randomly divided into two sets at a ratio of 3:1 using random number generation. The larger of the two sets, the ‘derivation set’, was used to produce a risk scoring model, which was then applied to the smaller ‘validation set’ to validate its predictive accuracy. To produce the risk score, each of the potential predictors was first considered univariably. For the categorical variables, the chi-squared test was used to compare rates of PM across the different levels of the factor. For continuous variables, Mann–Whitney *U* test was used. The factors found to be significant at this stage of the analysis were then entered simultaneously into a backwards stepwise binary logistic regression using the patients in the derivation set. The resulting model was then converted into a risk score using the log-odds values. To make these values more suitable for clinical use, they were multiplied by a constant. This score was then reapplied to the derivation set and a receiver operating characteristic (ROC) curve produced to ensure that it remained predictive of metastases. The risk score was then applied to the patients in the validation set and ROC curves again used to assess its predictive ability. All statistical analyses were performed using SPSS (IBM Corp., Armonk, NY).

## Results

**Figure 1:**
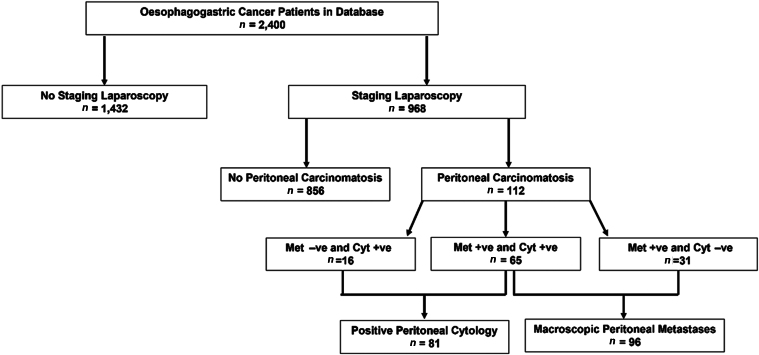
Flowchart detailing patients included and excluded for this study. Cyt +ve = positive peritoneal cytology; Cyt −ve = negative peritoneal cytology; Met +ve = peritoneal metastases present; Met −ve = peritoneal metastases absent

A total of 2,400 patients with OG cancer were identified from the institutional database. Of these, 1,432 patients were excluded because they did not undergo SL, either because they were not considered suitable for the curative pathway, for example because they had metastatic disease on staging CT scan, or they had contraindication to SL such as previous abdominal surgery. This left 968 patients who underwent SL and were included in the study. Ninety-six (9.9%) patients were found to have macroscopic PM and 81 (8.4%) were found to have PPC (Figure 1).

### Risk factors for peritoneal metastases

On univariate analysis, cancer site was a significant predictor of PM with gastric cancer having the highest rate of metastases at SL (*p* < 0.001). CT T and N stages were also significantly associated with metastases (*p* < 0.001 and *p* = 0.029 respectively). EUS stage, OGD histology, sex and age were not significantly associated with metastases (Table 1).

**Table 1:** Univariate analysis of factors associated with peritoneal metastases for all oesophagogastric cancer patients

	No metastases (*n* = 872)	Metastases (*n* = 96)	*p*-value
Age, median (IQR)	68 (60–74)	69 (60–77)	0.408
Male	550 (63)	57 (59)	0.768
Cancer site			**<0.001**
Oesophageal	280 (97)	8 (3)	
GOJ	367 (93)	29 (7)	
Gastric	225 (79)	59 (21)	
OGD histology			0.339
Adenocarcinoma	663 (91)	63 (9)	
Squamous cell qcarcinoma	57 (97)	2 (3)	
Other	65 (90)	7 (10)	
EUS T stage			0.462
T1–2	97 (25)	2 (25)	
T3	278 (71)	5 (63)	
T4	15 (4)	1 (13)	
EUS N stage			0.732
N0	163 (43)	2 (29)	
N1	194 (51)	5 (71)	
N2	18 (5)	0 (0)	
N3	4 (1)	0 (0)	
CT T stage			**<0.001**
T1	12 (100)	0 (0)	
T2	133 (98)	3 (2)	
T3	574 (90)	61 (10)	
T4	44 (71)	18 (29)	
CT N stage			**0.029**
N0	400 (92)	34 (8)	
N1	322 (91)	33 (9)	
N2	85 (89)	11 (12)	
N3	13 (72)	5 (28)	
PET			0.534
M0	579 (97)	26 (96)	
M1	16 (3)	1 (4)	

Values are given as *n* (%), unless noted otherwise.

CT = computed tomography; EUS = endoscopic ultrasound; GOJ = gastro-oesophageal junction; IQR = interquartile range; OGD = oesophagogastroduodenoscopy; PET = positron emission tomography

### Risk factors for positive peritoneal cytology

On univariate analysis, cancer site was a significant predictor of PPC with gastric cancer having the highest rate of metastases at SL (*p* < 0.001). CT T and N stages were also significantly associated with PPC (*p* < 0.001 and *p* < 0.001 respectively). There was a significant association between OGD histology and cytology, with no cases of positive cytology in the squamous cell carcinoma group. EUS staging, sex and age were not significantly associated with cytology (Table 2).

**Table 2:** Univariate analysis of factors associated with positive peritoneal cytology for all oesophagogastric cancer patients

	Negative cytology (*n*=885)	Positive cytology (*n*=81)	*p*-value
Age, median (IQR)	68 (59–74)	70 (61–77)	0.210
Male	487 (55)	47 (58)	0.312
Cancer site			**<0.001**
Oesophageal	283 (98)	8 (2)	
GOJ	368 (93)	29 (7)	
Gastric	234 (83)	59 (17)	
OGD histology			**0.041**
Adenocarcinoma	673 (93)	51 (7)	** **
Squamous cell carcinoma	59 (100)	2 (0)	
Other	64 (89)	8 (11)	
EUS T stage			0.096
T1–2	98 (99)	2 (1)	
T3	281 (99)	2 (1)	
T4	15 (94)	1 (6)	
EUS N stage			0.963
N0	163 (99)	2 (1)	
N1	197 (99)	2 (1)	
N2	18 (100)	0 (0)	
N3	4 (100)	0 (0)	
CT T stage			**<0.001**
T1	60 (97)	2 (3)	
T2	147 (99)	1 (1)	
T3	581 (92)	54 (9)	
T4	47 (77)	14 (23)	
CT N stage			**<0.001**
N0	407 (94)	25 (6)	
N1	327 (92)	28 (8)	
N2	89 (93)	7 (7)	
N3	11 (61)	7 (39)	
PET			0.118
M0	585 (97)	20 (3)	
M1	15 (88)	2 (12)	

Values are given as *n* (%), unless noted otherwise.

CT = computed tomography; EUS = endoscopic ultrasound; GOJ = gastro-oesophageal junction; IQR = interquartile range; OGD = oesophagogastroduodenoscopy; PET = positron emission tomography

### Generation of score to predict peritoneal metastases and positive peritoneal cytology

Significant variables from the univariate analyses (Tables 1 and 2) proceeded to multivariate analysis in the derivation set. The models produced were then applied to the derivation set and remained a significant predictor of both metastases and PPC as measured by ROC curves (data not shown).

Therefore, the model was then converted to a risk score format using the log-odds values for both PM and positive cytology. These were multiplied by a constant to make them more user-friendly and this is listed in Table 3 for PM and Table 4 for PPC**.**

**Table 3:** Values assigned for each variable in risk score for peritoneal metastases

	log-odds	Score value
Cancer site – Oesophageal		0
Cancer site – GOJ	2.061	4
Cancer site – Gastric	3.436	7
CT T stage = Tx		0
CT T stage = T1–2	0.002	0
CT T stage = T3	1.527	3
CT T stage = T4	2.383	5

GOJ = gastro-oesophageal junction

**Table 4:** Values assigned for each variable in risk score for positive peritoneal cytology

	log-odds	Score value
Cancer site – Oesophageal		0
Cancer site – GOJ	2.574	5
Cancer site – Gastric	3.433	7
CT T stage = Tx-2		0
CT T stage = T3	1.936	4
CT T stage = T4	2.742	5
CT N stage = N0		0
CT N stage = N1	0.620	0
CT N stage = N2	−0.047	0
CT N stage = N3	2.532	5

GOJ = gastro-oesophageal junction

**Figure 2:**
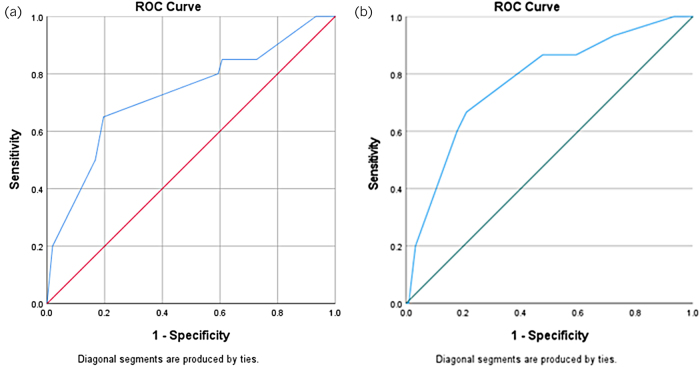
Receiver operating characteristic (ROC) curve for (a) peritoneal metastases risk score when tested on validation set and (b) positive peritoneal cytology risk score when tested on validation set.

These scores were then applied to the validation set and assessed using ROC curves***.*** This showed that both risk scores were significant predictors of their relevant outcome. For PM this is shown in Figure 2a (area under the receiver operating characteristic [AUROC] = 0.730; 95% confidence interval [CI] 0.601–0.859) and for PPC this is shown in Figure 2b (AUROC 0.773; 95% CI 0.649–0.897).

The rate of metastases for different score categories in the validation set is listed in Table 5. An increasing rate of metastases was observed as the score increased. Likewise, the rate of PPC for different score categories in the validation set is listed in Table 6, where once again, an increasing rate of metastases was observed as the score increased. An important example is early (T stage < 3 and N stage < 3) oesophageal tumours. The risk score for this group of patients was ‘0’ and appeared to be at very low risk of PM or PPC. If SL was not performed in the validation set of 228 patients based on this score, then 14 patients (6%) would have avoided SL.

**Table 5:** Rates of peritoneal metastases for different score values in the validation set

	Validation set (*n* = 228)	
Score	No metastases (*n* = 208)	Metastases (*n* = 20)
0	14 (100)	0 (0)
3	43 (93.5)	3 (6.5)
4	25 (100)	0 (0)
5	3 (75.0)	1 (25.0)
7	83 (96.5)	3 (3.5)
9	6 (66.7)	3 (33.3)
10	31 (83.8)	6 (16.2)
12	4 (50.0)	4 (50.0)

Values given as *n* (%)

**Table 6:** Rates of positive peritoneal cytology for different score values in the validation set

	Validation set (*n* = 228)	
Score	Negative cytology (*n* = 214)	Positive cytology (*n* = 15)
0	14 (100)	0 (0)
4	45 (97.8)	1 (2.2)
5	28 (96.6)	1 (3.4)
7	25 (100)	0 (0)
9	57 (95.0)	3 (5.0)
10	7 (87.5)	1 (12.5)
11	31 (83.8)	6 (16.2)
12–15	7 (70.0)	3 (30.0)

Values given as *n* (%)

## Discussion

This study aimed to create a risk score with preoperative factors that could be used at the time of OG cancer MDT to predict the risk of metastases and PPC at SL. The study demonstrated that a risk score using the CT T stage and site of malignancy is able to predict the presence of PM, and a risk score using the CT T and N stage with the site of malignancy is able to predict PPC at SL in OG cancer patients. Adopting these risk scoring systems into routine clinical practice will be useful and it may be possible to avoid unnecessary SL procedures (e.g. using a cut-off score of <4). However, even avoiding SL in those patients who score <4 still has a false-negative rate of 7%. From our data, it would be reasonable to conclude that all patients with gastric or GOJ tumours, as well as oesophageal tumours staged as T4 or N3, should have a laparoscopy. Oesophageal tumours of T1 or T2 staging with no lymph nodes (N0) do not require laparoscopy because these patients are at extremely low risk of having peritoneal disease. In the validation set, 6% of patients met these criteria and so SL could have been avoided. We have not reported the cost of SL in this study but others have reported this ranging between $20,000 and $100,000 per quality-adjusted life year.^22,23^ Indeed, one cost-effectiveness study from Li *et al* stresses the importance of performing SL in gastric cancer patients where the probability of metastatic disease is predicted to be high, around 31.5%.^22^ This is a good example of where a score such as the one reported here would be useful. Site of tumour alone was insufficient to predict the risk of peritoneal disease and our scoring system has the advantage of combining various factors (tumour site, T and N stage) into a risk stratification system. However, before clinical use in our MDTs and development of clinically relevant cut-offs can be determined, multicentre external validation of our score is required. Using the principles of our scoring system and performing laparoscopy only on those patients who need it could potentially avoid unnecessary delays to curative treatment, avoid wasting operating theatre capacity and prevent complications associated with the procedure such as port site hernias and wound infection.^11,12^ This is perhaps even more pertinent at the present time, as the Covid pandemic continues to place strain upon health systems worldwide and operating theatre capacity remains at a premium.

Evidence supports the use of SL in reducing the rate of unnecessary laparotomy in gastric cancer patients, where curative resection is attempted but not possible due to locally advanced or metastatic disease.^24-26^ In gastric cancer, this has a reported rate between 7.1% and 16.2%.^24,27^ This is actively avoided given the significant risk of perioperative morbidity and mortality as well as possible delays to chemotherapy while recovering from surgery.

Rates of PC reported in the literature range from around 45% in gastric cancer^10,11,13^ to 5%–11% in oesophageal cancer^9,13,14^ and, as expected, a greater rate of metastases was seen in gastric malignancies, followed by GOJ cancers then oesophageal malignancy in the current study. As such, the site of cancer was shown to be an independent predictor of metastases at SL and was incorporated into the risk score. It is important to acknowledge that there are some that advocate SL in all types of OG malignancy, including oesophageal and GOJ cancers,^13^ but the findings of the current, large study are clear and supported by other recent evidence that affirms the value of SL in preoperative staging in gastric cancer patients.^24,28^

In the current study, CT T stage was shown to be an independent predictor of metastases and positive cytology at SL and as such was incorporated into the risk score along with tumour location. This is consistent with findings reported elsewhere: with larger tumours shown to have greater rates of PC.^9–11^ Furthermore, a recent prospective study also showed a significant relationship between T stage and open/close surgery in gastric cancer patients, with tumours of T3–4 stage being a risk factor for unnecessary laparotomy.^24^

This study developed a risk score to predict for both PM and PPC. The reason for this is that the implications for these two patient groups are different. For example, the clinical relevance of PPC is not as clear cut as for PM; some believe those with positive cytology can be potentially converted in to potentially operable patients with a reasonable survival. This is mainly in patients who respond to chemotherapy, whose cytology becomes negative after chemotherapy response.^15,16^ Other units believe that the prognosis for cytology-positive disease should preclude surgical resection.^13^ There are also other complicating factors when comparing cytology with metastases; for example, peritoneal cytology has been reported to have a limited sensitivity for detecting peritoneal disease, which may be due in part to different cytology preparation and processing techniques across laboratories.^1,2,13^

### Study limitations

The limitations of this study are that the risk scores were produced using data from a single institution and the risk score has only been validated internally. Thus, for further evaluation of the efficacy of these risk scores, external validation should be performed. In addition, owing to the cohort of patients managed by our institution, there is a relatively large proportion of oesophageal cancer patients in the cohort. Furthermore, staging from any imaging modality, such as CT scans, is subjective and is therefore vulnerable to human error and bias. Although CT scans were reported by multiple consultant radiologists, these were specialist upper gastrointestinal radiologists who regularly attend the upper gastrointestinal MDT. Lastly, given that different cancer sites undergo different staging investigations preoperatively, although most patients had a CT scan there were a significant number who did not undergo EUS or PET scans. For example, at our institution, PET scans are mainly performed for patients with oesophageal cancer so it was not included in the current score. This is supported by recent evidence, which indicates that PET scans do not contribute much additional value in preoperative staging for gastric cancer.^28^ With multicentre collaboration, however, it may be possible to create a similar score for each of the individual cancer sites (oesophageal, GOJ and gastric). That said, this study includes a large number of patients, which was possible given that our institution is a high-volume centre with a prospectively collected and maintained database. This also meant that each of the patients followed a standardised pathway based on the latest clinical guidelines.^29^ The reason that many patients did not undergo EUS staging is likely to reflect the move to be more conservative with EUS staging, which does not appear to alter management in most patients. Current indications for EUS in our centre are fine needle aspiration sampling of out of field lymph nodes, suspected T4 disease, assessment of early tumours prior to attempted endoscopic resection or the assessment of margins in junctional tumours prior to deciding on the operative approach. Tumour differentiation could not be taken into account in the current study because preoperative biopsies from OGDs were used and there was an insufficient number of these biopsies reporting grade of differentiation for meaningful analysis.

## Conclusions

In conclusion, the current risk score is a valid tool with which to predict the risk of metastases at SL in OG cancer and may help to avoid subjecting patients to unnecessary SL. Although this scoring system may be a useful asset to decision-making in OG cancer MDTs, future studies with a focus on the role of more clinically applicable analysis could investigate clinical outcomes such as early recurrence and presence of micrometastases. This would be a useful area for future studies to focus on.
